# Zyflamend, a polyherbal mixture, down regulates class I and class II histone deacetylases and increases p21 levels in castrate-resistant prostate cancer cells

**DOI:** 10.1186/1472-6882-14-68

**Published:** 2014-02-21

**Authors:** E-Chu Huang, Yi Zhao, Guoxun Chen, Seung Joon Baek, Michael F McEntee, Steven Minkin, John P Biggerstaff, Jay Whelan

**Affiliations:** 1 Department of Nutrition, University of Tennessee, 1215 West Cumberland Avenue, Room 229 Jessie Harris Building, Knoxville, TN 37996, USA; 2 Department of Biomedical and Diagnostic Sciences, University of Tennessee, 2407 River Drive, Knoxville, TN 37996, USA; 3 Center for Environmental Biotechnology, University of Tennessee, Knoxville, TN 37996, USA; 4 Tennessee Agricultural Experiment Station, University of Tennessee, Knoxville, TN 37996, USA

**Keywords:** Zyflamend, p21, Epigenetic, Prostate cancer, CWR22Rv1, Histone deacetylase, HDAC, Histone acetyltransferase, CBP/p300, Herbs, Castrate-resistant

## Abstract

**Background:**

Zyflamend, a mixture containing extracts of ten herbs, has shown promise in a variety of preclinical cancer models, including prostate cancer. The current experiments were designed to investigate the effects of Zyflamend on the expression of class I and II histone deacetylases, a family of enzymes known to be over expressed in a variety of cancers.

**Methods:**

CWR22Rv1 cells, a castrate-resistant prostate cancer cell line, were treated with Zyflamend and the expression of class I and II histone deacetylases, along with their downstream target the tumor suppressor gene p21, was investigated. Involvement of p21 was confirmed with siRNA knockdown and over expression experiments.

**Results:**

Zyflamend down-regulated the expression of all class I and II histone deacetylases where Chinese goldthread and baikal skullcap (two of its components) appear to be primarily responsible for these results. In addition, Zyflamend up regulated the histone acetyl transferase complex CBP/p300, potentially contributing to the increase in histone 3 acetylation. Expression of the tumor suppressor gene p21, a known downstream target of histone deacetylases and CBP/p300, was increased by Zyflamend treatment and the effect on p21 was, in part, mediated through Erk1/2. Knockdown of p21 with siRNA technology attenuated Zyflamend-induced growth inhibition. Over expression of p21 inhibited cell growth and concomitant treatment with Zyflamend enhanced this effect.

**Conclusions:**

Our results suggest that the extracts of this polyherbal combination increase histone 3 acetylation, inhibit the expression of class I and class II histone deacetylases, increase the activation of CBP/p300 and inhibit cell proliferation, in part, by up regulating p21 expression.

## Background

The use of herbs, botanicals and their bioactive components have been shown to be effective in many tumor cell lines in vitro and in vivo by inhibiting cell and tumor growth. The use of herbal extracts in combination potentiates their actions, some synergistically, resulting in significant activity when the effects of any single agent are less robust [1,2]. Zyflamend (New Chapter, Inc., Brattleboro, VT) is a combination of the extracts of ten herbs, many of which are used as nutrient supplements (ginger, rosemary, turmeric, Chinese goldthread, holy basil, Hu Zhang, barberry, oregano, green tea and basil skullcap) (Table 1). It has been shown that Zyflamend has anticancer properties in experimental models of cancers, i.e., bone, skin, mouth, pancreas and kidney [3-8]. In addition, Zyflamend has been shown to reduce proliferation in a variety of prostate cancer (PrC) cell lines by modulating genes that impact the cell cycle and apoptosis [9-14]. Of particular interest to our laboratory is the effect of Zyflamend on castrate-resistant PrC [9,10,15].

**Table 1:** Composition of herbal extracts in Zyflamend and relative concentration of individual herbs used in vitro

**Compounds**	**Percentage (%)**	**Concentration (μg/mL) in 200 μg/mL Zyflamend**
Ginger	12.8	10.2
Rosemary	19.2	15.4
Turmeric	14.1	11.3
Chinese goldthread	5.1	4.1
Holy Basil	12.8	10.2
Hu Zhang	10.2	8.2
Barberry	5.1	4.1
Oregano	5.1	4.1
Green tea	12.8	10.2
Basil skullcap	2.5	2.0
Total	99.7	79.8

Histone deacetylases (HDACs) are a family of enzymes associated with cancer risk. Post-translational modification of histones, in particular the removal or addition of acetyl groups on ϵ-N-acetyl lysine residues, play an important role in epigenetic regulation of transcription. Acetylation of the N-terminal tails of histones “relaxes” the chromatin making it more accessible for binding by co-activating factors. The result is an increase in gene expression. In contrast, deacetylation results in a more compact chromatin and transcriptional repression. Regulation of acetylation is a balance between deacetylators and acetylators [16]. HDACs in particular are important in cancer biology by promoting proliferation, angiogenesis, migration/metastasis, resistance to chemotherapy, and inhibiting apoptosis and differentiation (as reviewed in Ref [17]). Identification of HDAC inhibitors is therefore a new therapeutic approach to treat cancer [18,19].

Eighteen different isoenzymes of HDACs have been identified and are divided into 4 classes, I-IV. Class I and II HDACs form complexes with multiple cofactors for activation where histones are a primary substrate [20] and have been targets for cancer therapies, including PrC [21]. They appear to be particularly important in regulating cell survival and proliferation [20,21]. Class I HDACs (types 1, 2, 3, 8) are located almost exclusively in the nucleus. Class II HDACs are subdivided where IIa (types 4, 5, 7, 9) has an N-terminal domain that regulates shuttling between the nucleus and cytoplasm. Class IIb HDACs (types 6, 10) are predominantly cytoplasmic and their functions are less well established. In castrate-resistant PrC cells, HDAC1 is overexpressed compared with androgen sensitive PrC cells [22] and HDAC4 is predominantly expressed in the nucleus of hormone refractory cancer cells [23], while HDAC8 does not appear to be expressed in PrC epithelial cells [24]. HDACs 1–4 have been shown to be involved in the repression of p21 expression [25-27]. HDAC6 is unique in that it contains two catalytic domains that independently contribute to its activity. HDAC6 is predominately found in the cytoplasm whose major substrates include α-tubulin and Hsp90 [28-30]. HDAC6 over expression has been associated with a variety of cancer cell lines, including prostate [30]. Class III HDACs (SIRT1-7) also require a unique set of cofactors for activity that are distinctly different from those involved with class I and II HDACs. They are NAD-dependent, share homology to yeast Sir 2 family of deacetylases and their primary targets are not histones [16]. HDAC11 (class IV) is structurally related to class I and II HDACs, but little is known about this HDAC.

The goal of this project was to better understand the properties of the anticancer effects of the combination of bioactives from Zyflamend. Our previous research demonstrated that Zyflamend, when provided orally, inhibited tumor growth using a xenograph model of castrate-resistant PrC in vivo and these effects were associated with inhibition of expression of HDACs 1 and 4 [9]. To better understand the effects of Zyflamend on HDAC expression, we followed up our in vivo results by investigating the broader effects of Zyflamend on the expression of class I and II HDACs in the same model of castrate-resistant PrC.

Prostate cancer is currently the most commonly diagnosed solid malignancy and has become the second leading cause of cancer-related deaths in men in most Western developed countries [31]. One in six men will develop invasive prostate cancer in their lifetime [31]. Metastatic PrC is defined as the spread of PrC cells to secondary sites (i.e., bone, lung, etc.). Once tumors become metastatic, they are very difficult to treat, and prognosis is poor with a 31% 5-year survival rate [32]. For the most part, PrC is temporarily responsive to hormone deprivation therapy as prostate epithelial cells are dependent on androgens for growth. While treatment with hormone deprivation results in tumor regression and clinical stabilization, the disease eventually relapses, with invariable fatal results within two years. Therefore, a critical barrier in treating advanced PrC is finding effective adjuvant treatments for castrate-resistant forms of the disease. The CWR22Rv1 PrC cell line was chosen for the experiments because it represents a late stage of PrC [33] and our preliminary experiments using this cell line in vivo linked Zyflamend treatment with HDAC inhibition [9]. These cells can grow in the presence or absence of androgens, produce prostate specific antigen (PSA) and express a functional androgen receptor [33]. These critical factors are consistent with PrC in patients whose disease has relapsed following androgen ablation therapy as their tumors can grow in the absence of androgens, typically have functional androgen receptors and can produce PSA.

In this study, we investigated the effects of Zyflamend on expression of class I and class II HDACs and downstream targets, such as the tumor suppressor gene p21. This work was designed to explore some of the molecular mechanisms behind the anti-carcinogenic effects of Zyflamend. This study was not designed to compare Zyflamend with the pharmacokinetics of a variety of commercially known HDAC inhibitors, although Zyflamend was compared to the general HDAC inhibitor trichostatin A (TSA).

## Methods

### Zyflamend

Zyflamend (New Chapter, Brattleboro, VT) is derived from the extracts of ten different herbs (w/w): holy basil *(Ocimum sanctum)* (12.8%), turmeric *(Curcuma longa)* (14.1%), ginger *(Zingiber officinale)* (12.8%), green tea *(Camellia sinensis)* (12.8%), rosemary *(Rosmarinus officinalis)* (19.2%), Hu Zhang *(Polygonum cuspidatum)* (10.2%), barberry *(Berberis vulgaris)* (5.1%), oregano *(Origanum vulgar)* (5.1%), baikal skullcap *(Scutellaria baicalensis)* (2.5%), and Chinese goldthread *(Coptis chinensis)* (5.1%). The total portion of extracts in Zyflamend is 40% (Table 1). A detailed description and characterization of the preparation of Zyflamend and quality assurance of the mixture has been described previously [9].

### Cell culture

Human prostate cell lines, RWPE-1, LNCaP, PC3 and CWR22Rv1, were purchased from American Type Culture Collection (Rockville, MD). PrEC cells (Lonza, Walkerville, MD) were grown in Clonetics™ Bulletkit™ medium according to the supplier’s instructions. RWPE-1 cells were maintained in complete medium containing keratinocyte serum free medium supplemented with bovine pituitary extract (BPE) (0.05 mg/mL) and human recombinant epidermal growth factor (hEGF) (5 ng/mL). LNCaP and PC3 cells were maintained in RPMI 1640 media (Life Technologies, Grand Island, NY) supplemented with 10% fetal bovine serum (FBS) (Life Technologies, Grand Island, NY) under an atmosphere of 5% CO_2_ at 37°C. Cells were harvested with the addition of 0.25% trypsin with 0.02% EDTA during the exponential growth phase. For the experimental treatments, CWR22Rv1 cells were cultured in RPMI 1640 media supplemented with 0.05% fetal bovine serum containing Zyflamend or individual herbal extracts (ginger, rosemary, turmeric, Chinese goldthread, holy basil, Hu Zhang, barberry, green tea and baikal skullcap) (each supplied by New Chapter, Inc, Brattleboro, VT) reconstituted in dimethyl sulfoxide (DMSO) for cell proliferation assay, mRNA extraction and protein isolation. For inhibitor experiments, CWR22Rv1 cells were pretreated with U0126 (Erk inhibitor) (Cell Signaling Technology, Inc., Danvers, MA) at a dose of 2 μM for 30 minutes and subsequently treated with Zyflamend (200 μg/mL) for 24 hr. For experiments involving the general HDAC inhibitor TSA, TSA was added to CWR22Rv1 cells at a concentration of 2 μM for 24 hours and compared to cells treated with Zyflamend (200 μg/mL). In all experiments, 0.1% DMSO was used as the vehicle control.

### Cell proliferation

The MTT assay [3-(4, 5-dimethylthiazol-2-yl)-2, 5-diphenyltetrazolium bromide] (Andwin Scientific, Addison, IL) was used to assess relative cell growth and viability, following the manufacturer’s instructions (Promega, Madison, WI). Cells (1 × 10^4^ cells of RWPE-1, LNCaP, and CWR22Rv1, and 5 × 10^3^ cells of PC3) were plated in 96-well plates in a volume of 100 μl culture medium. The culture medium contained various concentrations of Zyflamend (0, 40, 80, 100, 150, 200 μg/mL) or individual herbal extracts (10.2 μg/mL ginger, 15.4 μg/mL rosemary, 11.3 μg/mL turmeric, 4.1 μg/mL Chinese goldthread, 10.2 μg/mL holy basil, 8.2 μg/mL Hu Zhang, 4.1 μg/mL barberry, 10.2 μg/mL green tea, 2.0 μg/mL baikal skullcap; equivalent to those doses in 200 μg/mL Zyflamend, Table 1). Cell proliferation was determined at 0, 24, 48, 72, 96 hr post incubation. At each time point, a mixture of MTT:complete medium (1:10, v/v) was added and incubated at 37°C for 4 hr in a CO_2_ incubator (5%). Absorbance (at 540 nm) was measured on a SpectraCount microplate photometer (Perkin Elmer Inc, Waltham, MA).

### BrdU incorporation assay

Cells (1 × 10^4^ cells of CWR22Rv1) were plated in 96-well plates and treated with various concentrations (0, 100, 150 and 200 μg/mL) of Zyflamend for 48 hr and followed by a BrdU incorporation assay to assess relative DNA synthesis following the manufacturer’s instructions (EMD Biosciences, Inc., Darmstadt, Germany). After Zyflamend treatment, cells were treated with BrdU for 4 hr and the BrdU incorporation was measured on a FluoroCount microplate photometer (Perkin Elmer Inc, Waltham, MA) at a 340 nm excitation and a 460 nm emission.

### Cellular and nuclear detection of p21 via immunofluorescent imaging

CWR22Rv1 cells were seeded on cover slips in RPMI 1640 media supplemented with 10% FBS under an atmosphere of 5% CO_2_ at 37°C overnight. Before the treatment, CWR22Rv1 cells were maintained in RPMI 1640 media with 0.5% FBS. For the observation of p21 and its nuclear localization, the cells were pretreated with Zyflamend (200 μg/mL) for 24 hr. After the treatment, the cells were fixed using 2% paraformaldehyde for 15 min, followed by blocking with 10% goat serum (Santa Cruz Biotechnology, Inc., Santa Cruz, CA) for 1 hr, and anti-p21 antibody (10 μg/mL) (Santa Cruz Biotechnology, Inc., Santa Cruz, CA) overnight at 4°C. After washing with PBS, coverslips were incubated with secondary antibody (10 μg/mL of Alexa Fluor 488 goat anti-rabbit IgG) for one hour at room temperature. Coverslips were mounted on glass slides with Prolong Gold w/ DAPI Antifade reagent (Invitrogen Corporation, Carlsbad, CA) and analyzed by epifluorescence microscopy. Four dual-channel images were captured from each sample using a 60x objective lens. Image analysis was performed using NIS-Elements software v3.1 (Nikon Instruments, Melville, NY). Mean fluorescence intensity per cell was calculated by the following: [(total p21 fluorescence)/(nuclei count)]. To assess p21 nuclear accumulation, p21 fluorescence was also measured within discrete nuclear regions as defined using a DAPI intensity threshold.

### Down regulation of p21 by small interfering RNA

CWR22Rv1 (1.5 × 10^5^ cells per well in 6-well plates, in serum-free RPMI 1640 media) were transfected with validated p21 small interfering RNA (siRNA) or Stealth™ siRNA negative control (Invitrogen, Carlsbad, CA) (100 pmole each) using Lipofectamine 2000™ transfection reagent (Invitrogen, Carlsbad, CA) following the manufacturer’s instruction. Six hr post transfection, cells were cultured with RPMI 1640 media containing 10% FBS overnight. After recovery, media was replaced with 0.05% FBS media containing vehicle or Zyflamend (200 μg/mL) for 24 hr at 37°C. The total RNA was harvested for quantitative real-time polymerase chain reaction (qRT-PCR) and cell number was determined.

### Overexpression of p21

pRc/CMV-p21 (Addgene Inc., Cambridge, MA), containing full length wild-type p21 cDNA, was used to overexpress p21. CWR22Rv1 cells (1.5 × 10^5^ cells per well in 6-well plates) were plated overnight. pRc/CMV-p21 or pRc/CMV (empty vector) was transfected using Lipofectamine 2000™ reagent in serum-free RPMI 1640 media. Transfected cells were selected by treatment for two weeks with neomycin (50 μg/mL) and subjected to the MTT cell proliferation assay. p21 protein expression in the transfected cells was examined by Western blot.

### RNA isolation and quantitative RT-PCR

Total RNA was isolated from CWR22Rv1 cells using Trizol reagent (Invitrogen, Carlsbad, CA) followed by chloroform extraction. The aqueous phase was precipitated in 100% isopropanol and the pellet was washed in 75% ethanol prior to re-suspension in RNase-free water. Contaminating DNA was removed from RNA samples using Turbo DNA free™ kit (Ambion, Inc. Austin, TX) and then the concentration of total RNA was measured using NanoDrop 1000™ (Thermo Scientific, Wilmington, DE). Total RNA (2 μg) from each sample was mixed with MultiScribe™ Reverse Transcriptase, RNase Inhibitor, dNTP Mixture, random hexamers, RT buffer, MgCl_2_ solution and incubated at 25°C for 10 min, 48°C for 30 min and 95°C for 5 min to reverse transcribe to cDNA using TaqMan reagent kit (Applied Biosystems, Carlsbad, CA). cDNA samples were used for quantitative RT-PCR (ABI Prism 7300 Real-Time PCR System, Applied Biosystems, Carlsbad, CA). cDNA (0.7 μl) was used as a template for qPCR amplification with primer sets (2.5 μM) of p21 sense, 5′-TGGAGACTCTCAGGGTCGAAAA-3′ and antisense, 5′- CCGGCGTTTGGAGTGGTA-3′, p27 sense, 5′-CGGTGGACCACGAAGAGTTAA-3′ and antisense, 5′-GGCTCGCCTCTTCCATGTC-3′, HDAC1 sense, 5′-ACCGGGCAACGTTACGAAT-3′ and antisense, 5′-CTATCAAAGGACACGCCAAGTG-3′, H DAC2 sense, 5′-TCATTGGAAAATTGACAGCATAGT-3′ and antisense, 5′-CATGGTGATGGTGTTGAAGAAG-3′, HDAC3 sense, 5′- TTGAGTTCTGCTCGCGTTACA-3′ and antisense, 5′-CCCAGTTAATGGCAATATCACAGAT-3′, HDA4 sense, 5′-AATCTGAACCACTGCATTTCCA-3′ and antisense, 5′-GGTGGTTATAGGAGGTCGACACT-3′, HDAC5 sense, 5′-TTGGAGACGTGGAGTACCTTACAG-′3 and antisense 5′-GACTAGGACCACATCAGGTGAGAAC-3′, HDAC6 sense, 5′-TGGCTATTGCATGTTCAACCA-3′ and antisense 5′-GTCGAAGGTGAACTGTGTTCCT-3′, HDAC7 sense, 5′-CTGCATTGGAGGAATGAAGCT-3′ and antisense 5′-CTGGCACAGCGGATGTTTG-3′, were examined. Amplification was performed using a standard thermocycle program beginning with an initial temperature at 94°C for 1 min followed by 30 cycles of 94°C for 15 sec, 50°C for 30 sec and 72°C for 2 min. Each sample was examined in triplicate and the amounts of PCR product were normalized with 36B4, 5′-TGCATCAGTACCCCATTCTATCA-3′ and 5′-AAGGTGTAATCCGTCTCCACAGA-3′ as the internal control. The relative amounts of all mRNAs were calculated using the comparative *C*_*T*_ method as previously described with 36B4 as the invariant control [34,35]. The relative amounts of 36B4 and the various transcripts were calculated using the following formula: relative amounts of mRNA = 1/2^(*CT*-*Time X* - *CT*-Time 0)^, where C_*T-Time X*_ is the *C*_*T*_ number at one experiment time point, and *C*_*T-Time 0*_ is the *C*_*T*_ number at time 0. The levels of 36B4 and the various transcripts at time 0 were arbitrarily assigned as 100%.

### Protein degradation

CWR22Rv1 cells were cultured with RPMI 1640 medium containing in the presence and absence of Zyflamend (200 μg/mL) for 24 and 48 hr to demonstrate induction of p21 expression. Cells were also exposed to Zyflamend for 24 hr and then maintained for another 24 hr in the absence of Zyflamend. In addition, cells were treated with Zyflamend (200 μg/mL) for 24 hr prior to adding cycloheximide (10 μM) to terminate protein synthesis for an additional 0, 0.5, 1, 1.5, 2, 4 hr in the continued presence or absence of Zyflamend (200 μg/mL) and then harvested for protein analysis.

### Western blotting

CWR22Rv1 cells were lysed in the presence of cell lysis buffer (Cell Signaling Technology, Inc. Danver, MA). Protein content of the lysates was quantified by BCA protein assay kit (Pierce Biotechnology, Inc., Rockford, IL). Lysates (20 μg) were fractioned by 8 ~ 12% SDS-PAGE and transferred to a polyvinylidine diflouride (PVDF) membrane by electroblotting. The membranes were blocked using 5% nonfat dry milk in 0.1% Tris-buffered saline-Tween-20 (TBST) for 1 hour at room temperature and incubated in TBST containing primary antibodies overnight at 4°C. The membrane was incubated with anti-mouse or anti-rabbit secondary antibody conjugated with horseradish peroxidase (HRP) (Cell Signaling Technology, Inc. Danver, MA). Protein expression was detected with a Pierce ECL Western Blotting detection system. Each membrane was exposed to Hyperfilm Film (GE Healthcare, Piscataway, NJ). Antibodies of p21, p27, p53, HDAC1-7, Erk, phospho-Erk (Cell Signaling Technology, Inc. Danver, MA) were used. β-actin (with anti-β-actin antibody, Santa Cruz Biotechnology, Inc., Santa Cruz, CA) was used as the control.

### HDAC activity assay

CWR22Rv1 cells were lysed in the presence of cold lysis buffer (10 mM Tris–HCl, pH 7.5, 10 mM NaCl, 15 mM Mg_2_Cl_2_, 250 mM sucrose, 0.5% NP-40, and 0.1 mM EGTA). Cytosolic and nuclear protein fractions were isolated through NE-PER™ Nuclear and Cytoplasmic Extraction Reagents (Pierce Biotechnology, Rockford, IL) following manufacturer’s instructions and HDAC activity assays (Cayman Chemical, Ann Arbor, MI) were performed as per manufacturer’s instructions. The assay was measured using an excitation wavelength of 340 nm and an emission wavelength of 460 nm (FluoroCount, Perkin Elmer Inc, Waltham, MA).

### Statistical analysis

The results are presented as mean ± SEM and the mRNA results are presented as mean ± SD. For two group comparisons, the data was analyzed by two-tailed Student’s T-statistic. For multiple comparisons, the results were analyzed by an ANOVA followed by Tukey’s post hoc analysis when appropriate. Differences were considered significant at p < 0.05.

## Results

### Prostate cancer cell growth and DNA synthesis are inhibited by Zyflamend

**Figure 1:**
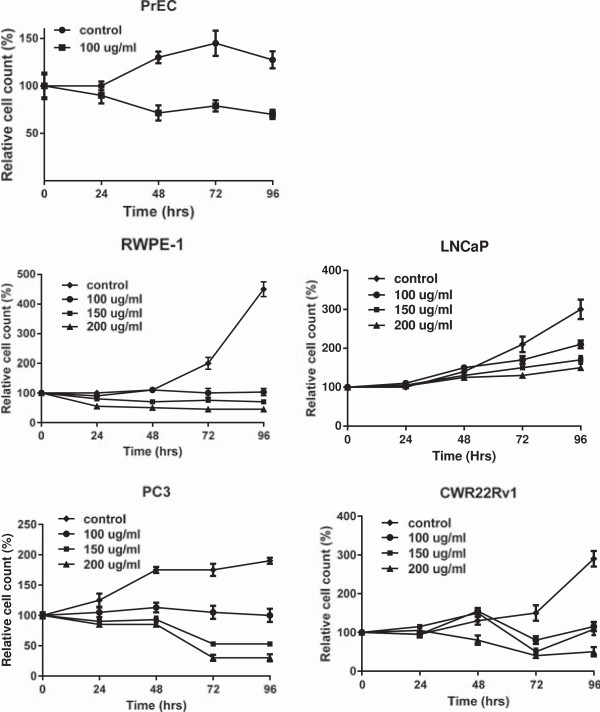
**Zyflamend’s effects on cell proliferation of PrEC, RWPE-1, LNCaP, PC3 and CWR22Rv1 cells using MTT assay.** Cells were treated ± Zyflamend at various concentrations (0, 100, 150 and 200 μg/mL) over 24, 48, 72 and 96 hr (n = 8/time period).

**Figure 2:**
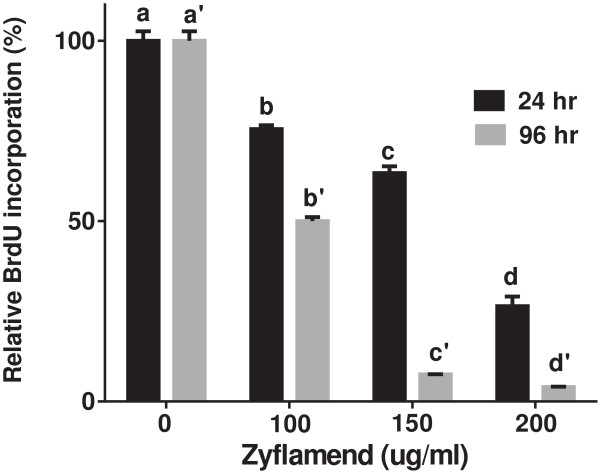
**Effects of Zyflamend on BrdU incorporation in a time- and dose-dependent manner.** Cells were treated ± Zyflamend at various concentrations (0, 100, 150 and 200 μg/mL) over 24 and 96 hr (n = 8/time period).

Zyflamend inhibited growth of all PrC cell lines tested in a time and concentration-dependent manner. At the end of 96 hr treatment, Zyflamend (200 μg/ml) inhibited cell growth in PrEC cells by 45%, RWPE-1 cells by 80%, LNCaP cells by 60%, PC3 cells by 50% and CWR22Rv1 cells by 75% (Figure 1). To further confirm the reduction of cell proliferation of CWR22Rv1 cells by Zyflamend, BrdU assay was used for determining DNA synthesis during the cell cycle. After treatment with Zyflamend (24 and 96 hr), BrdU incorporation in CWR22Rv1 cells was reduced in a time and concentration-dependent manner (Figure 2).

### Zyflamend inhibits expression of HDACs

In the presence of Zyflamend, mRNA expression of all HDACs tested (HDAC 1–7) was reduced by 30-80%, and HDAC activity (cytosolic and nuclear extracts) was inhibited (Table 2). When cells were treated with individual herbal extracts, only Chinese goldthread and baikal skullcap appeared to mimic the down-regulation of mRNA observed with Zyflamend with regards to all HDACs tested (Table 2). The effects of the extracts of rosemary, Hu Zhang, holy basil, turmeric, green tea, barberry and ginger were more variable by having mixed effects on HDAC expression. Rosemary appeared to upregulate mRNA for HDAC4 and down regulate HDAC6, turmeric upregulated HDACs 1, 4, and 7, barberry down-regulated HDAC2 and upregulated HDAC5, holy basil upregulated HDACs 1 and 4 and down-regulated HDAC6, green tea upregulated HDAC7 and down-regulated HDACs 2 and 3 and ginger upregulated HDACs 4, 5 and 7 and down-regulated HDAC2. Protein levels of HDACs 1, 2, 4 and 7 were significantly reduced following treatment with Zyflamend (Table 3). The universal HDAC inhibitor TSA recapitulated the results of Zyflamend on HDAC expression and cell proliferation (Table 3).

**Table 2:** Effects of individual herbal extracts or Zyflamend® on mRNA expression of class I and II histone deacetylases and relative HDAC activity compared to controls

	**Control**	**Zyflamend**	**Rosemary**	**Turmeric**	**Barberry**	**Hu Zhang**	**Gold Thread**	**Skull Cap**	**Holy Basil**	**Green Tea**	**Ginger**
	Relative mRNA (n = 3)^1^										
HDAC1	1.00 ± 0.04	0.42 ± 0.05*	0.76 ± 0.07	1.28 ± 0.07*	0.94 ± 0.04	0.92 ± 0.02	0.57 ± 0.05*	0.76 ± 0.01*	1.61 ± 0.02*	1.22 ± 0.13	0.95 ± 0.03
HDAC2	1.00 ± 0.01	0.34 ± 0.03*	0.79 ± 0.10	0.94 ± 0.15	0.67 ± 0.07*	0.70 ± 0.05	0.35 ± 0.04*	0.23 ± 0.02*	1.09 ± 0.04	0.87 ± 0.02*	0.52 ± 0.06*
HDAC3	1.00 ± 0.02	0.17 ± 0.02*	0.94 ± 0.05	0.99 ± 0.06	0.58 ± 0.17	1.09 ± 0.02	0.26 ± 0.01*	0.35 ± 0.03*	0.82 ± 0.09	0.69 ± 0.05*	0.91 ± 0.07
HDAC4	1.00 ± 0.10	0.13 ± 0.01*	1.80 ± 0.09*	1.84 ± 0.08*	1.56 ± 0.25	1.07 ± 0.05	0.59 ± 0.04*	0.47 ± 0.02*	1.52 ± 0.12*	1.35 ± 0.10	2.63 ± 0.11*
HDAC5	1.00 ± 0.06	0.63 ± 0.06*	1.21 ± 0.24	1.40 ± 0.25	1.34 ± 0.05*	1.18 ± 0.09	0.27 ± 0.35*	0.61 ± 0.02*	1.12 ± 0.13	1.16 ± 0.12	1.54 ± 0.14*
HDAC6	1.00 ± 0.03	0.20 ± 0.01*	0.70 ± 0.05*	0.81 ± 0.12	1.16 ± 0.10	1.02 ± 0.05	0.27 ± 0.01*	0.38 ± 0.02*	0.84 ± 0.03*	0.72 ± 0.13	1.22 ± 0.09
HDAC7	1.00 ± 0.16	0.39 ± 0.04*	1.18 ± 0.02	1.64 ± 0.04*	1.41 ± 0.01	1.10 ± 0.04	0.53 ± 0.09*	0.61 ± 0.03*	1.37 ± 0.09	1.47 ± 0.02*	1.78 ± 0.06*
	Relative HDAC activity (n = 3), cellular location:				Cytosolic		Nuclear				
					Control	Zyflamend	Control	Zyflamend			
					1.00 ± 0.05	0.72 ± 0.08*	1.00 ± 0.08	0.75 ± 0.02*			

^1^All data is relative to control values. Relative mRNA and HDAC activity data are expressed as mean ± SEM.

^*^Values with an asterisk are significantly different from control at p < 0.05. *Abbreviation*: HDAC, Histone deacetylase.

**Table 3:** Effects of Zyflamend or TSA on cell proliferation, protein expression of various histone deacetylases and p21 (relative to controls)

**Compounds**	**Zyflamend (200 ug/ml)**	**TSA (2 μM)**
HDAC1	0.49 ± 0.08*	0.39 ± 0.06*
HDAC2	0.64 ± 0.10*	0.93 ± 0.06
HDAC3	1.14 ± 0.13	0.88 ± 0.17
HDAC4	0.56 ± 0.12*	0.38 ± 0.01*
HDAC7	0.62 ± 0.05*	0.68 ± 0.09*
p21	2.38 ± 0.39*	6.05 ± 0.65*
Relative cell proliferation	0.53 ± 0.11*	0.45 ± 0.04*

Values are mean ± SEM; n = 5/group. ^*^Values with an asterisk are significantly different from control at p < 0.05. *Abbreviations*: HDACs, histone deacetylase; TSA, trichostatin A.

### Zyflamend-mediates the induction of cell cycle inhibitors p21 and p27, mRNA and protein

**Figure 3:**
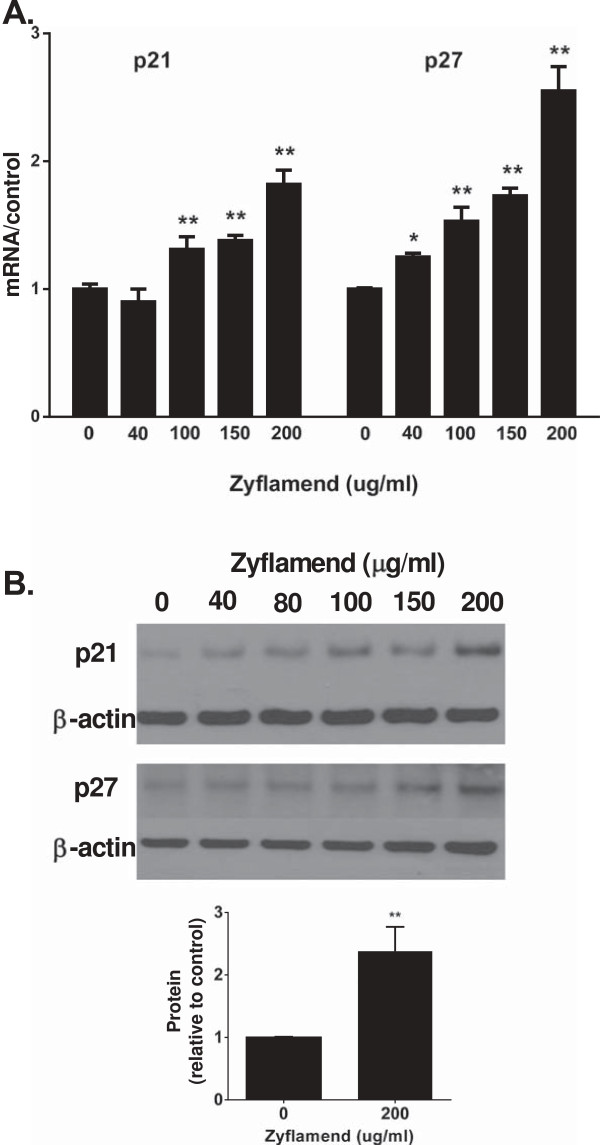
**Impact of Zyflamend on mRNA and protein expression of p21 and p27.** Cells were treated with increasing concentrations of Zyflamend (0, 40, 100, 150 and 200 μg/mL). **(A)** Expression of mRNA levels. **(B)** Expression of protein levels. The bar graph quantitates the relative protein level of p21 ± Zyflamend at 0 and 200 μg/mL. mRNA and protein values are mean ± SEM, n = 3. Values are significantly different from non-treatment: *p < 0.05; **p < 0.01.

**Figure 4:**
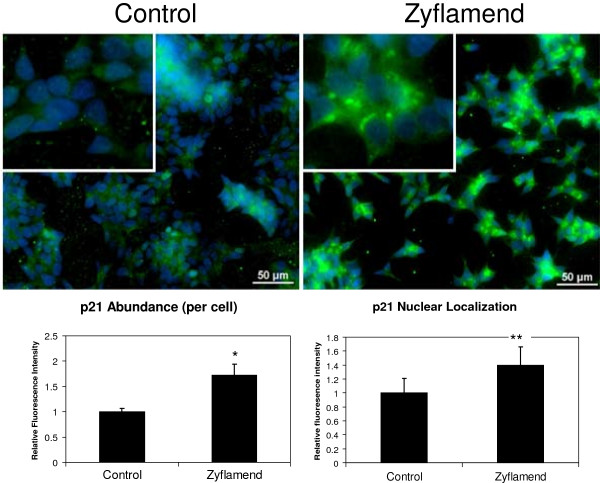
**Zyflamend and its effects on cellular p21 expression as determined by immunofluorescent imaging using CWR22Rv1 cells.** Cells pretreated with 200 μg/ml Zyflamend for 24 hours (right) were compared with control cells (left). Nuclei are shown in blue. Mean p21 (green) abundance per cell was calculated [(total p21 fluorescence)/(nuclei count)]. To assess nuclear accumulation, p21 fluorescence was also measured within discrete nuclear regions (defined using a DAPI intensity threshold). Data are presented as the mean ± SD in relative fluorescence units. Representative images feature magnified inlays.* p < 0.001, n = 4; **p < 0.001, n = 112.

In CWR22Rv1 cells, Zyflamend treatment induced mRNA levels for the cell cycle inhibitors p21 and p27 (Figure 3A). Concomitantly, protein levels of p21 were increased by as much as 2.4-fold with Zyflamend treatment compared to control (Figure 3B). While p27 levels also were increased, we focused our attentions on p21 due to the robust nature of the results and the literature linking phytonutrients with p21 expression [36]. Our results were supported by immuno-fluorescent imaging. 4′, 6-diamidino-2-phenylindole (DAPI), a blue fluorescent stain that binds strongly to DNA, was used to label nuclei. The intensity of green fluorescent staining is an indication of relative p21 protein levels. It is clear from the imaging panels that Zyflamend increased p21 levels per cell (+72%) and increased nuclear accumulation (+40%) (Figure 4). Changes in p21 protein levels were related to increased expression and not by inhibiting protein turnover based on experiments using cycloheximide (Additional file 1). The HDAC inhibitor TSA also increased p21 expression (Table 3).

### p21 silencing induces cell growth

**Figure 5:**
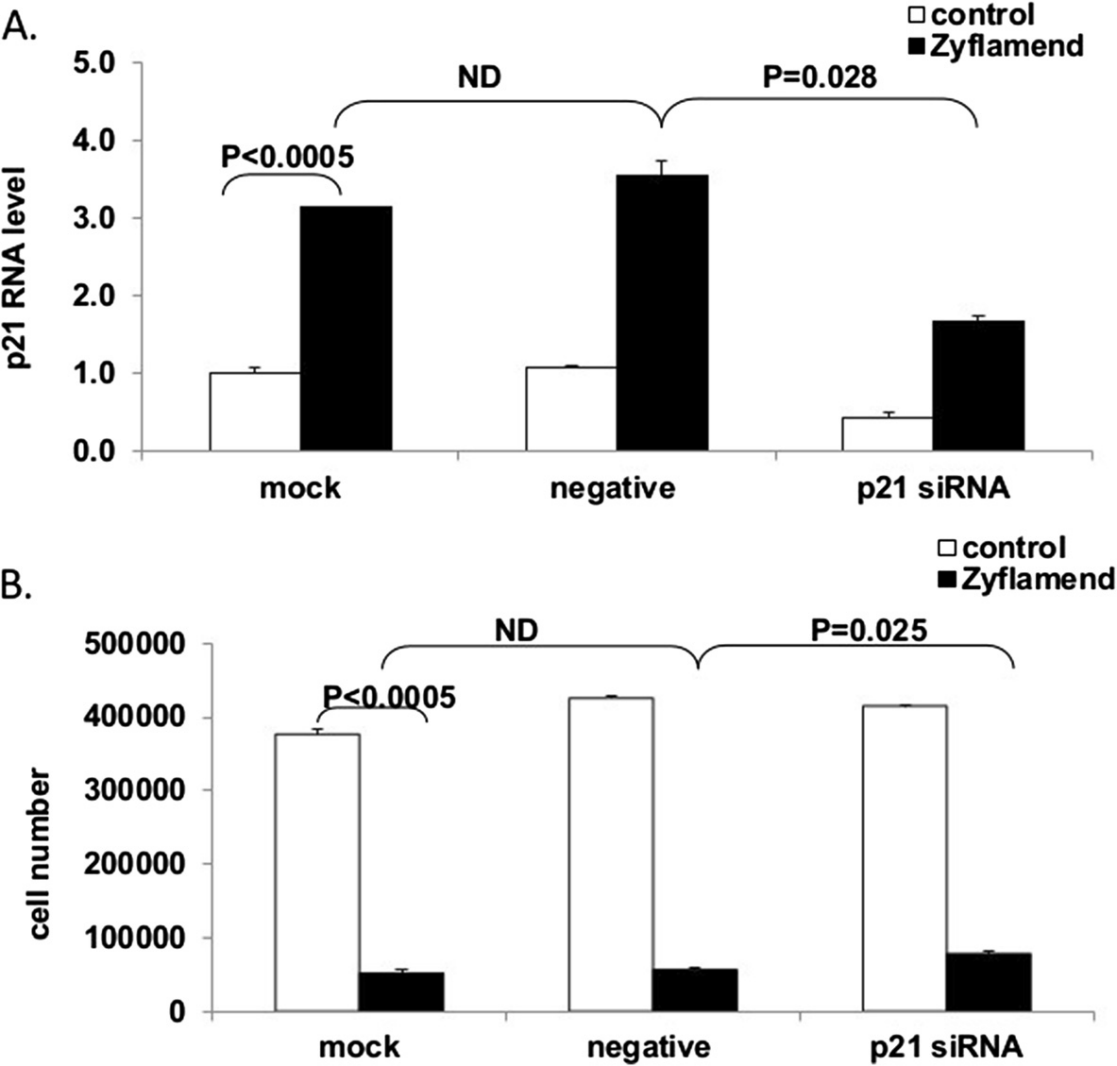
**Impact of modifying p21 expression on cell growth in the presence or absence of Zyflamend using p21 silencing.** p21 mRNA levels **(A)** and cell proliferation **(B)** were determined in the presence or absence of Zyflamend (24 or 96 hr, respectively) in cells transfected with mock (reagent only), negative (scramble siRNA) and p21 siRNA. Data are presented as the mean ± SD (n = 3).

CWR22Rv1 cells were transfected with siRNA against p21 in the presence or absence of Zyflamend. Zyflamend increased p21 mRNA expression in mock (p < 0.005) and in negative control siRNA transfections (p < 0.05) (Figure 5A) with concomitant reductions in cell number (Figure 5B). Transfection of p21 siRNA reduced p21 mRNA in the absence or presence of Zyflamend (Figure 5A). Comparing the mock/negative control groups to the p21 siRNA group in the presence of Zyflamend (closed bars), there was a reduction in p21 mRNA levels with p21 siRNA treatment (Figure 5A) and a concomitant increase (~51%) in cell number (Figure 5B). However, in cells not treated with Zyflamend (open bars), cell numbers did not change following p21 siRNA treatment (Figure 5B) despite reduced p21 expression below the baseline, suggesting basal levels of p21 are not regulating proliferation.

### p21 overexpression reduces cell growth

**Figure 6:**
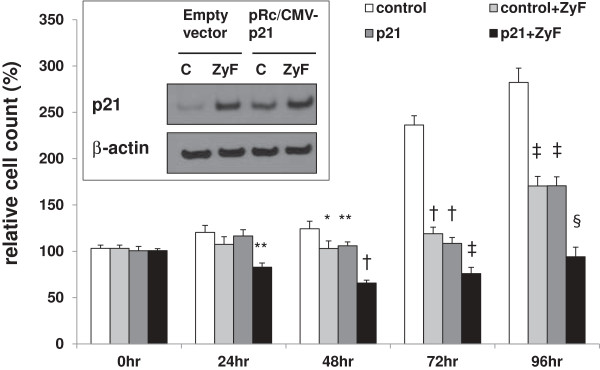
**Effects of the overexpression of p21 on cell growth in the presence or absence of Zyflamend.** CWR22Rv1 cells were transfected with pRc/CMV (empty vector) or pRc/CMV-p21 and treated in the presence or absence of Zyflamend for 24 hr. Relative expression of p21 (protein) is presented in the insert. Following transfection with the empty vector (pRc/CMV) or pRc/CMV-p21 (p21), cells were treated ± Zyflamend for 0–96 hr. Cell proliferation was determined using the MTT assay. Control, open bar, Control + Zyflamend (Zyf), light grey bar; Control + pRc/CMV-p21 (p21), dark grey bar; Zyflamend + pRc/CMV-p21 (p21), black bar. Data are presented as the mean ± SEM. *p < 0.05, **p < 0.005*,* †p < 0.0005, ‡p < 0.00005 and §p < 0.000005; (n = 8).

To mimic the effect of the induction of p21 by Zyflamend, p21 was overexpressed in CWR22Rv1 cells and confirmed by Western blot (Figure 6 insert). Both p21 overexpression and the presence of Zyflamend reduced cell proliferation over time (p < 0.0005) (Figure 6). The reduction of cell proliferation by p21 overexpression was potentiated in the presence of Zyflamend (p < 0.00005). These results were supported, in part, by the fact that Zyflamend increases p21 promoter activation (data not shown) using a human p21 promoter (2.4 kb) luciferase reporter construct, consistent with increases in mRNA and protein levels.

### Zyflamend-induces Erk1/2, histone 3 acetylation and acetyl CBP/p300 expression

**Figure 7:**
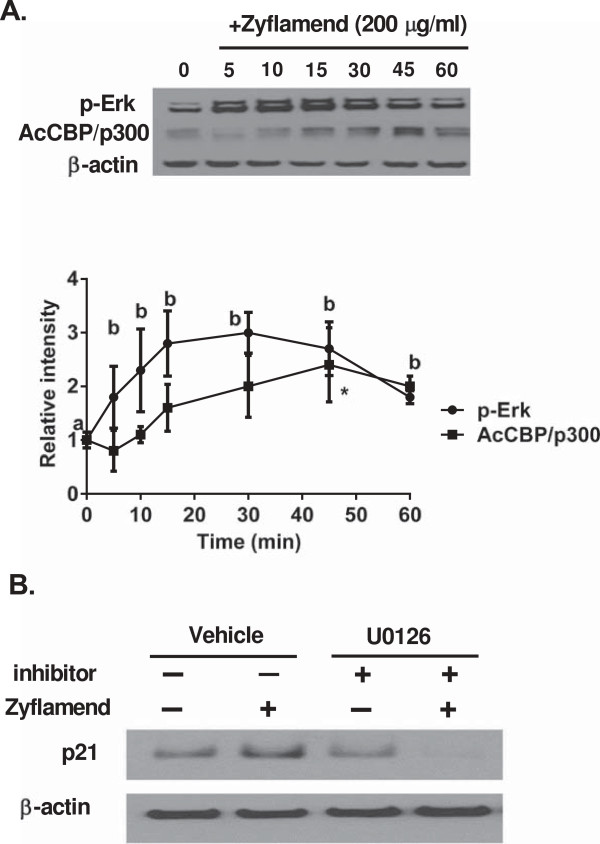
**Zyflamend’s effects on protein expression of pErk and acetyl-CBP/p300 in a time dependent manner, and on p21 expression ± Zyflamend in the presence or absence of the HDAC inhibitor U0126. (A)** CWR22Rv1 cells were treated with Zyflamend (200 μg/mL) and the expression of p-Erk and acetyl-CBP/p300 was plotted over time (0–60 min, n = 3 for each time point). The data was analyzed by ANOVA. Data points with different letters (p-Erk) are different at p < 0.05. With regards to Ac-CBP/p300, the only data point different from t = 0 is identified with an asterisk (*). Data are presented as the mean ± SD. **(B)** Western blot of p21 in CWR22Rv1 cells treated ± Zyflamend (200 μg/mL) in the presence or absence of the HDAC inhibitor U0126 (2 μM) for 24 hr.

**Figure 8:**
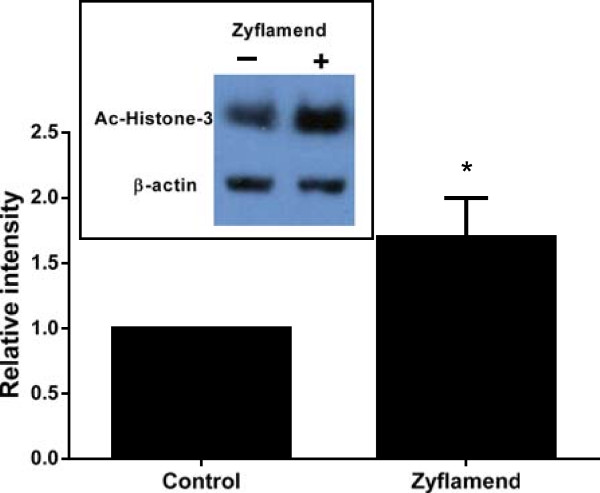
**Zyflamend increases histone 3 acetylation.** Protein level of acetylated histone 3 was determined in CWR22Rv1 cells treated ± Zyflamend (200 μg/ml) for 24 hr. * denotes significantly different from control at p < 0.05. Data are presented as the mean ± SD, n = 3.

CBP/p300 are transcriptional co-activators that have histone acetyl transferase activity (as reviewed by Marmorstein and Trievel [37]), and it has been reported that CBP/p300 are downstream targets of extracellular signal-related kinase (Erk) [38]. Zyflamend increased the levels of phosphorylated Erk and acetylated CBP/p300 (Figure 7) in a time-dependent manner with the levels of pErk increasing prior to the increase of Ac-CBP/p300. To investigate the involvement of mitogen-activated protein (MAP) kinases on Zyflamend-induced p21 protein expression, we used the Erk inhibitor U0126, an inhibitor that selectively targets Erk activity without inhibiting p38 or c-Jun N-terminal kinase (JNK). U0126 reduced Zyflamend-induced p21 levels (Figure 7). Since HDACs and CBP/p300 activities affect the structure of chromatin by modifying histone acetylation and thus transcriptional expression of target genes such as p21, histone acetylation was examined. Histone 3 acetylation was significantly increased in the presence of Zyflamend (Figure 8).

## Discussion

The use of herbs and botanicals and their bioactive components are effective inhibitors of growth, angiogenesis, metastasis and inducing apoptosis in many tumor cell lines. Many of their molecular mechanisms of action have been characterized in vitro [2,39,40]. While the use of combinations of bioactive compounds appear to potentiate each other’s actions [1,2], not much data exists with herbal extracts in combination as would be common in cultures where botanicals are used as medicinal therapies.

We previously reported that Zyflamend inhibited the proliferation of castrate-resistant PrC cells in vitro*,* and growth of androgen dependent and castrate-resistant derived PrC tumors in vivo [9,10]. We also reported that Zyflamend inhibited the expression of insulin-like growth factor-1 receptor (IGF-1R) and androgen receptor in CWR22Rv1 cells in vitro [10]. Inhibition of androgen receptor expression was recapitulated using CWR22Rv1-derived tumors in mice treated orally with Zyflamend [9]. This is important because up regulation of IGF-1R and androgen receptor signaling has been linked to relapse of PrC following hormone ablation therapy [41,42]. To broaden the growing literature on the effects of Zyflamend, we also reported that Zyflamend inhibited HDAC expression (1 and 4) in xenograph models of androgen-dependent and castrate-resistant PrC (in vivo), and wanted to further investigate its impact on the expression of class I and II HDACs and one of their reported targets the tumor suppressor gene p21 [9].

Zyflamend inhibited the growth of PrEC, RWPE-1, LNCaP and PC3 prostate cell lines, in addition to the castrate-resistant PrC cell line CWR22Rv1. With regards to PrEC and RWPE-1 prostate cells, the results on growth inhibition by Zyflamend are novel, while those observed with LNCaP, PC3 and CWR22Rv1 cells are consistent with results published previously [10,11], thus validating our current results. Similar to the results presented here, all cell lines tested, in addition to normal and non-tumorigenic prostate epithelial cells (PrEC and RWPE-1cells, respectively), have previously been shown to be sensitive (inhibition of proliferation) to polyphenolics, flavonoids and various botanical extracts [43-50]. PrEC cells represent a normal prostatic epithelial cell line and RWPE-1 cells are a non-tumorigenic human prostate epithelial cell line transfected with the human papilloma virus-18. LNCaP cells are an androgen-dependent PrC tumor cell line; while PC3 cells are androgen-independent (PC3 cells do not have a functional androgen receptor and cannot produce PSA). Because of our interest in castrate-resistant PrC, we focused our attention on CWR22Rv1 cells.

Over expression of various forms of HDACs is a characteristic of PrC and is associated with shorter relapse times [51], and development of castrate-resistant PrC has been linked to upregulation and nuclear localization of the androgen receptor [52]. Zyflamend recapitulated and expanded upon part of our earlier work [9] by down regulating the expression of all HDACs tested. In addition to HDACs 1 and 4, the down regulation of HDAC6 is of particular interest because HDAC6 mediates nuclear translocation of the androgen receptor via deacetylation of Hsp90 in castrate-resistant PrC cells [30]. In this study, Zyflamend decreased HDAC6 expression and concomitantly Zyflamend also decreased the expression and nuclear localization of the androgen receptor [10]. These new data contribute to a growing number of pathways impacted by Zyflamend, helping to explain its multiple mechanisms of action. In an effort to identify which extracts contributed most to the effects on inhibition of HDAC expression, we observed that Chinese goldthread and baikal skullcap recapitulated the results observed with Zyflamend. While we cannot rule out synergistic/antagonistic actions by the other extracts in the preparation, these data suggest that Chinese goldthread and baikal skullcap are most likely the major contributors inhibiting HDAC expression by Zyflamend.

Treatment of CWR22Rv1 cells with Zyflamend resulted in increased acetylation of histone 3, a key feature of HDAC inhibitors. Epigenetic regulation via acetylation is important in regulating tumor suppressor genes, and p21 is a common target for bioactive phytonutrients [36,53]. Zyflamend consistently enhanced mRNA and protein levels of p21 (including the nuclear fraction) in dose- and time-dependent manners and these effects were recapitulated by the general HDAC inhibitor TSA (positive control). Importantly, when Zyflamend was added to cells overexpressing p21, there was an added reduction in cell proliferation, further suggesting the effects of Zyflamend do not rely solely on p21 expression, but potentially involve multiple mechanisms [3,5,8,11,14]. HDACs have been shown to be important upstream regulators of p21 [54], and hyperacetylation of Sp1 binding sites in the proximal promoter is a key regulator of p21 expression [54]. HDAC1 and HDAC4 have been reported to repress p21 expression [22,25,26,54-56]. Nuclear localization of HDAC4 is enhanced in human tissues of castrate-resistant PrC [23] and HDAC4 has been shown to regulate p21 expression through a Sp1-dependent, p53-independent pathway [26].

The effects on histone 3 acetylation led us to also investigate the potential upregulation of histone acetyl transferase activity [57] because of our findings that Zyflamend upregulated the activation of Erk1/2. The histone acetyltransferase activity of CBP/p300 can be regulated upstream by Erk1/2 and its downstream regulator, Elk-1 [38,58]. Erk1/2-dependent phosphorylation of Elk-1 results in interaction with p300 and increased histone acetyltransferase activity [38]. In a time-dependent manner, Zyflamend increased the expression of pErk, followed by CBP/p300 activation [59], where it appeared that Erk1/2 phosphorylation preceded the activation of CBP/p300. Inhibition of Erk1/2 using the Erk inhibitor U0126 attenuated Zyflamend-induced p21 levels. Stimulation of p21 expression via upregulation of the Erk pathway has been observed by others and these effects were similarly blocked in the presence of the Erk1/2 inhibitor U0126 [60]. While CBP/p300 has been linked to p21 expression [61,62], we have yet to fully characterize CBP/p300’s involvement in these cells. Furthermore, while CBP/p300 has been reported as a tumor suppressor [63], others report opposite findings [64] as these effects maybe tumor specific.

## Conclusions

In summary, Zyflamend, which is composed of ten concentrated herbal extracts, inhibited the growth of CWR22Rv1 cells in vitro, in part, by upregulating the tumor suppressor protein p21. These effects occurred concomitantly with histone acetylation, a known activator of p21 expression and cell cycle regulator. Increased expression of p21 occurred in concert with down regulation of class I and class II HDACs where Chinese goldthread and baikal skullcap may have the greatest effects, along with up regulation of pErk signaling and concomitant activation of CBP/p300. These data, in addition to the data previously published in castrate-resistant PrC cells (in vitro and in vivo), suggest a polyherbal mixture may have utility in helping to treat advanced forms of PrC.

## Abbreviations

CBP: CREB-binding protein; FBS: Fetal bovine serum; HDAC: Histone deacetylase; HRP: Horseradish peroxidase; MAP kinase: Mitogen-activated protein kinase; PrC: Prostate cancer; PVDF: Polyvinylidene fluoride; PSA: Prostate specific antigen; SDS-PAGE: Sodium dodecyl sulfate polyacrylamide gel electrophoresis; TBST: Tris-buffer saline tween-20; TSA: Trichostatin A; ZyF: Zyflamend.

## Competing interests

The following co-authors have no conflicts of interest: E.–C. Huang, G. Chen, S.J. Baek, S. Minkin, J. Biggerstaff, Y. Zhao and M.F. McEntee. All authors have received no reimbursements, fees, or salary from any organization associated with this research; nor do they hold any stocks or shares or in any way could gain financially from the publication of this manuscript now or in the future. They have not applied for and do not hold any patents related to the content of the work. They have not served in any capacity on administrative or scientific advisory boards. This research was supported, in part, by a grant from New Chapter, Inc, Brattleboro, VT (J. Whelan) where Zyflamend was provided at no cost.

## Authors’ contributions

E–CH and JW had primary responsibility for design of research; E–CH conducted research, with help from YZ; E–CH and JW co-wrote manuscript; JW had primary responsibility for the final content of the research and manuscript; SB and GC provided methodological expertise; SB, GC and MFM provided research and design support. SM and JB provided immunofluorescent imaging support. SB, GC, SM and MFM provided contributions to the manuscript. All authors approved final manuscript.

## Pre-publication history

The pre-publication history for this paper can be accessed here:

http://www.biomedcentral.com/1472-6882/14/68/prepub

## Supplementary Material

Additional file 1 **Zyflamend® increases p21 protein synthesis but does not prevent degradation.** A) p21 protein expression was monitored in the presence or absence of Zyflamend® (200 ug/ml) over 48 hrs. CWR22Rv1 cells were treated with Zyflamend® for 24 hrs (+24) after which time the cells were treated for an additional 24 hrs (+48) or in the absence of Zyflamend® for an addition 24 hrs (-48) and p21 expression was monitored. B) p21 protein expression was monitored in the presence of ±Zyflamend® for 24 hrs (0 hr time point). p21 protein levels were monitored for an additional 4 hrs ±Zyflamend in the presence of cyclohexamide. Click here for file
