# Supplementary material for: Zyflamend, a polyherbal mixture, down regulates class I and class II histone deacetylases and increases p21 levels in castrate-resistant prostate cancer cells
Source: BMC Complement Altern Med. 2014 Feb 21;14:68. doi: 10.1186/1472-6882-14-68 (PMC3938081; doi:10.1186/1472-6882-14-68)
Supplement: Additional file 1 — Zyflamend® increases p21 protein synthesis but does not prevent degradation. A) p21 protein expression was monitored in the presence or absence of Zyflamend® (200 ug/ml) over 48 hrs. CWR22Rv1 cells were treated with Zyflamend® for 24 hrs (+24) after which time the cells were treated for an additional 24 hrs (+48) or in the absence of Zyflamend® for an addition 24 hrs (-48) and p21 expression was monitored. B) p21 protein expression was monitored in the presence of ±Zyflamend® for 24 hrs (0 hr time point). p21 protein levels were monitored for an additional 4 hrs ±Zyflamend in the presence of cyclohexamide. [file 1472-6882-14-68-S1.pdf]

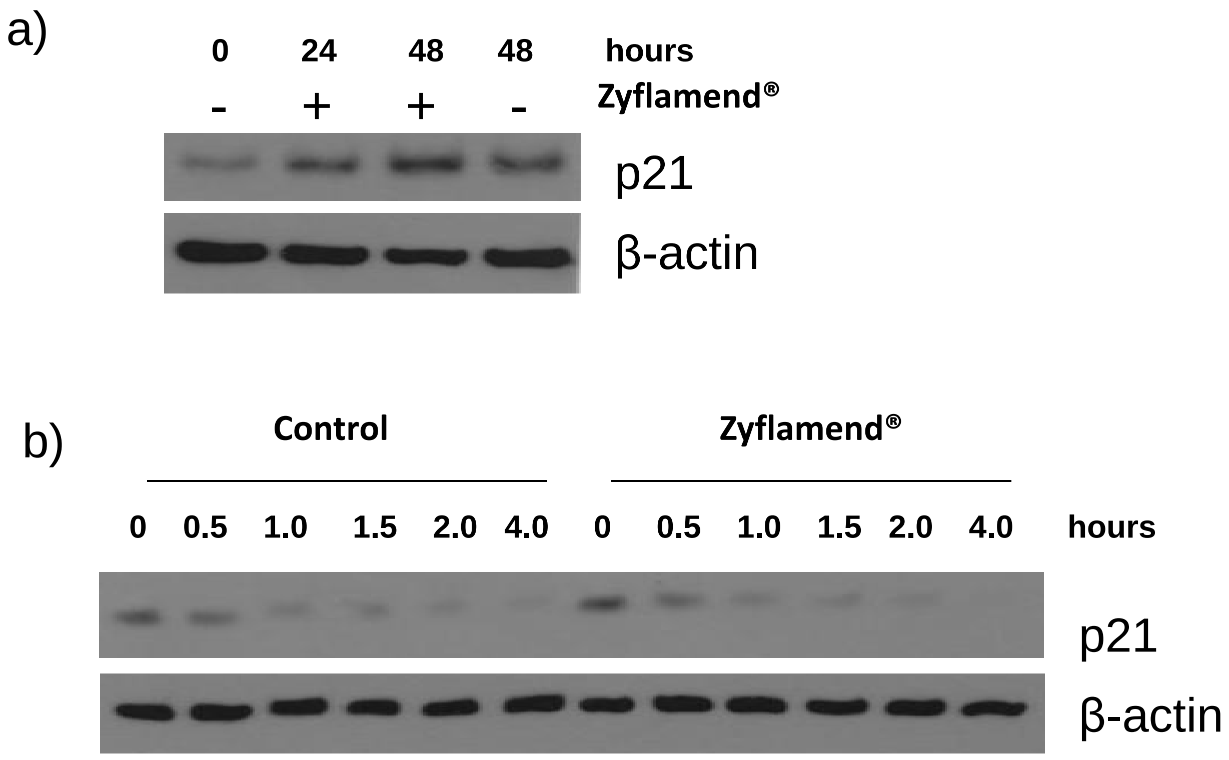

Additional File 1: Zyflamend® increases p21 protein synthesis but does not prevent degradation. A) p21 protein expression was monitored in the presence or absence of Zyflamend® (200 ug/ml) over 48 hrs. CWR22Rv1 cells were treated with Zyflamend® for 24 hrs (+24) after which time the cells were treated for an additional 24 hrs (+48) or in the absence of Zyflamend® for an addition 24 hrs (-48) and p21 expression was monitored. B) p21 protein expression was monitored in the presence of ±Zyflamend® for 24 hrs (0 hr time point). p21 protein levels were monitored for an additional 4 hrs ±Zyflamend in the presence of cyclohexamide.
